# Eigene Krisen- und Behandlungserfahrungen unter psychiatrischem Fachpersonal – Bedeutung und Umgang

**DOI:** 10.1007/s00115-025-01815-9

**Published:** 2025-03-14

**Authors:** Anna Brieger, Angel Ponew, Christian Lust, Sven Speerforck, Stefan Stützle, Sebastian von Peter

**Affiliations:** Hochschulklinik für Psychiatrie und Psychotherapie, an der Immanuel Klinik Rüdersdorf, Medizinische Hochschule Brandenburg Theodor Fontane, Seebad 82/83, 15562 Rüdersdorf, Deutschland

**Keywords:** Peer- und Genesungsbegleitung, Stigmatisierung, Selbstoffenbarung, Patient*innenbezogene Arbeit, Psychiatrische Fachpersonen, Peer- and recovery support, Self disclosure, Stigmatization, Psychiatric professionals, Patient-centered work

## Abstract

**Hintergrund:**

Eigene Krisen- und Behandlungserfahrungen (EKB) sind unter Mitarbeitenden in psychiatrischen und psychosozialen Bereichen weit verbreitet. Das vorliegende Teilprojekt des EKB-Forschungsprojektes, das die Betroffenheit von Mitarbeitenden psychiatrischer Einrichtungen in Berlin und Brandenburg untersucht, wendet sich den Fragen zu, welche Bedeutung diese Mitarbeitenden ihren EKB in der patient*innenbezogenen Arbeit beimessen und wie sie zu einer Offenlegung dieser Erfahrungen stehen.

**Material und Methoden:**

In einer explorativen, querschnittlichen Onlinebefragung wurden 182 psychiatrische Fachpersonen nach der Offenlegung ihrer EKB im Arbeitskontext befragt. Die Daten wurden deskriptiv und analytisch ausgewertet.

**Ergebnisse:**

Das Vorhandensein von EKB wird von den befragten Mitarbeitenden überwiegend positiv bewertet und als einflussreich für die Arbeit und nützlich für Patient*innen angesehen. Die Offenlegung dieser Erfahrungen wird neutral bis negativ beurteilt und selten praktiziert.

**Diskussion:**

Mitarbeitende zögern, ihre EKB offenzulegen. In Anlehnung an Literatur zum Thema der Peer- und Genesungsbegleitung ist zu vermuten, dass EKB durch reflektierte Offenlegung mehr Nutzen in der patient*innenbezogenen Arbeit bringen könnte.

**Zusatzmaterial online:**

Zusätzliche Informationen sind in der Online-Version dieses Artikels (10.1007/s00115-025-01815-9) enthalten.

## Abstract (en)

**Background:**

Own experiences of crises and treatment (ECT) are widespread among staff in psychiatric and psychosocial settings. The present subproject of the ECT research project, investigating how staff of psychiatric institutions in Berlin and Brandenburg are affected, addresses the questions of what significance these employees attach to the ECT in patient-related work and how they feel about disclosing these experiences.

**Material and methods:**

In an explorative cross-sectional online survey, 182 mental health professionals were asked about the disclosure of the ECTs in the work context. The data were analyzed descriptively and analytically.

**Results:**

The existence of ECT is predominantly rated positively by the staff surveyed and is seen as influential for their work and useful for patients. The disclosure of these experiences was assessed as neutral to negative and rarely practiced.

**Discussion:**

Staff are reluctant to disclose their ECT. Based on the literature on the topic of peer and recovery counselling, it can be assumed that ECT could bring more benefits in patient-related work through reflective disclosure.

## Einführung

Trotz des zunehmenden Interesses an den psychischen Problemen von Fachpersonal in Gesundheitsberufen [22, 25] gibt es nur wenige empirische Untersuchungen zu diesem Thema [3, 23, 30]. Grice et al. [11] fanden heraus, dass 67 % der befragten angehenden Psycholog*innen im Vereinigten Königreich über eigene psychische Gesundheitsprobleme berichteten. Eine Umfrage unter US-amerikanischem Fachpersonal (*n* = 264) der psychiatrischen Versorgung ergab, dass 75 % der Teilnehmenden eine Vorgeschichte mit psychischen Erkrankungen hatten [12]. In einer Umfrage unter israelischem Fachpersonal (*n* = 128) erfüllten 81 % der Teilnehmer die DSM-IV(Diagnostic and Statistical Manual of Mental Disorders, 5th Edition)-Kriterien für psychische Störungen [19].

Eigene Krisen- und Behandlungserfahrungen sind also keine Seltenheit unter Mitarbeitenden psychiatrischer oder psychosozialer Arbeitsfelder. Dabei umfasst der in der vorliegenden Studie gewählte Begriff „eigene Krisen- und Behandlungserfahrungen“ (EKB) ein breites Spektrum an persönlichen Erlebnissen, die mit psychischen Krisen und/oder deren Behandlung verbunden sind. Dazu zählen unter anderem Erfahrungen mit psychischen Erkrankungen, emotionalen oder sozialen Krisen, die entweder ärztlich, therapeutisch oder auch ohne externe Unterstützung bewältigt wurden. Für das Versorgungssystem in Deutschland lagen bis vor Kurzem kaum Daten zur Frage vor, wie häufig eigene Krisen- und Behandlungserfahrungen unter Mitarbeitenden psychiatrischer Einrichtungen vorkommen und wie sie damit umgehen.

Die vorliegende Arbeit ist Teil eines umfassenden Forschungsprojekts, das zwischen 2017 und 2020 die Häufigkeit und den Umgang mit EKB bei Mitarbeitenden psychiatrischer Kliniken der Pflichtversorgung in Berlin und Brandenburg untersuchte [26]. Im Rahmen der Studie wurden 218 Fachpersonen aus 18 Abteilungen der psychiatrischen Grundversorgung aus den Bundesländern Berlin und Brandenburg mittels Onlinebefragung einbezogen. Untersucht wurden persönliche Krisenerfahrungen, die Inanspruchnahme von Hilfe, Einstellungen zur Offenlegung sowie die Bedeutung dieser Erfahrungen für die berufliche Identität. Über 80 % der Teilnehmenden berichteten von psychischen Krisen, häufig in Form depressiver Episoden. Rund 70 % gaben an, ihre erste Krise bereits vor dem Berufseinstieg erlebt zu haben, und viele berichteten von mehreren Krisen. In knapp 50 % der Fälle führten diese Erfahrungen zu Suizidgedanken oder Arbeitsunfähigkeit. Ein Viertel der Befragten hatte Erfahrungen mit der Einnahme von Psychopharmaka gemacht, während drei Viertel bereits eine psychiatrische, psychosoziale oder psychotherapeutische Behandlung in Anspruch genommen hatten. Obwohl viele Fachpersonen ihre EKB als wertvoll für ihre Arbeit wahrnahmen, scheuten sie häufig vor einer Offenlegung zurück, was auf die Angst vor Stigmatisierung und möglichen beruflichen Konsequenzen zurückzuführen war.

Im Rahmen der EKB-Studie ist auch eine Arbeit zur Frage der Offenlegung von EKB gegenüber Kolleg*innen veröffentlicht worden [28, 29]. Diese zeigt, dass die untersuchten Mitarbeitenden diese Erfahrungen selten offenlegen, v. a. aufgrund von Befürchtungen, sich angreifbar zu machen und ihre berufliche Identität zu gefährden sowie aus Scham. Im Anschluss daran nutzt die vorliegende Arbeit Datenmaterial zur Offenlegung und zum zugeschriebenen Nutzen von EKB im Rahmen der patient*innenbezogenen Arbeit. Sie folgt dabei den drei Forschungsfragen, 1) welche Bedeutung psychiatrische Fachpersonen ihren EKB in der patient*innenbezogenen Arbeit beimessen, 2) ob sie diese Erfahrungen gegenüber Patient*innen offenlegen und 3) wie sie zu dieser Offenlegung stehen.

## Methoden

### Stichprobe

Für die vorliegende Publikation wurden Daten aus der EKB-Onlineumfrage ausgewertet, die von Mai bis September 2020 stattfand [29]. Neben der Untersuchung der Häufigkeit von EKB unter psychiatrischem Fachpersonal verschiedener Berufsgruppen wurden Fragen zum Umgang mit Offenlegung und empfundenen Stigma gestellt, zum Selbstverständnis der Mitarbeitenden und zu ihrer beruflichen Sozialisation sowie zu dem vermuteten Nutzen der EKB im Hinblick auf die Behandlung der Patient*innen. Die Studie verwendete eine Gelegenheitsstichprobe, wobei die Teilnehmenden über interne Kommunikationskanäle der psychiatrischen Kliniken in Berlin und Brandenburg auf die Onlineumfrage aufmerksam gemacht wurden. Der für die Studie entwickelte Fragebogen wurde in mehreren Schritten erstellt: Basierend auf einer umfassenden Literaturrecherche und Expert*inneninterviews wurden relevante Themenbereiche identifiziert und entsprechende Fragen formuliert. Der Fragebogen wurde von Fachleuten auf Verständlichkeit und Relevanz geprüft, um sicherzustellen, dass alle wichtigen Aspekte abgedeckt sind. Weitere Details zur Methodik und den Ergebnissen sind in der Hauptpublikation der EKB-Studie verfügbar [26].

### Datenerhebung

Um einer Stigmatisierung entgegenzuwirken, wurde im Rahmen dieser Untersuchung EKB mittels eines weit gefassten Krisenbegriffs erfasst. Die Teilnehmenden wurden gefragt, ob sie jemals eine „psychische Beanspruchung und oder Einschränkung des gewohnten ‚Funktionierens‘“ erlebt haben, inklusive „einmaliger Belastungsereignisse und länger anhaltender Zustände psychischer Beschwerden, die sich auch im Rahmen psychischer Störungsbilder gruppieren lassen“. Diejenigen Teilnehmenden, die EKB angaben, wurden um weitere Angaben dazu gebeten (u. a. Häufigkeit, Art, Nutzung verschiedener Hilfeangebote) – Ergebnisse, die im Detail anderenorts veröffentlicht wurden [28, 29].

Die wahrgenommene Bedeutung von EKB für die psychiatrische (Patient*innen‑)Arbeit wurde mithilfe von Items erfasst, die sich inhaltlich aus Literatur zum Nutzen von Genesungs- und Peer-Begleitung [10, 21, 31] und zu Mitarbeiter*innen mit EKB [11, 18, 22, 23] speisten. Zwei übergreifende Items fragten nach einer generellen Einschätzung der Auswirkungen von EKB bei Mitarbeitenden auf Patient*innen („Eigene Krisen‑/Behandlungserfahrungen bei Profis haben einen Einfluss auf die Arbeit mit Patient*innen“ und „Es nützt Patient*innen, wenn Profis über eigene Krisen‑/Behandlungserfahrungen verfügen“). Dann wurden insgesamt zehn positive und negative Zuschreibungen gegenüber dieser Mitarbeitenden-Gruppe präsentiert (z. B. „Profis mit EKB vermitteln Hoffnung“ oder „Profis mit EKB sind nicht ausreichend belastbar“). Eine vollständige Liste der verwendeten Items findet sich im Ergebnisteil. Die Teilnehmenden wurden gebeten, ihre Zustimmung auf einer 5‑stufigen Likert-Skala anzugeben (0 = keine Zustimmung, 4 = volle Zustimmung).

Die Einstellung der Teilnehmenden zur Offenlegung wurde mithilfe eines Items („Es ist sinnvoll, wenn Profis gegenüber PatientInnen ihre EKB offen thematisieren“) und mit einer 5‑stufigen Likert-Skala erfasst (0 = keine Zustimmung, 4 = volle Zustimmung).

Die Offenlegungspraxis, also die tatsächliche Offenlegung der eigenen EKB gegenüber Patient*innen, wurde mittels eines Items erfragt („Ich habe meine EKB – in welcher Form und zu welchem Zweck auch immer – gegenüber Patient*innen offenbart“). Es standen sechs Antwortmöglichkeiten in aufsteigender Häufigkeit zur Verfügung („noch nie“, „einmalig“, „mehrmals“, „regelmäßig“, „häufig“ und „immer [gegenüber allen Patient*innen]“).

Die Ethikkommission der Medizinischen Hochschule Brandenburg hielt ein ethisches Genehmigungsverfahren für nicht erforderlich, da die Population der Studie weder zu einer gefährdeten Gruppe gehöre noch ein Abhängigkeitsverhältnis zu berücksichtigen sei. Gleichzeitig wurde ein umfangreiches Datenschutzverfahren umgesetzt, dem alle Teilnehmenden zugestimmt haben.

### Analyse

Die Daten wurden zunächst deskriptiv ausgewertet. Anschließend wurde eine explorative Korrelationsanalyse (Pearson-Korrelationen) aller erhobenen Variablen durchgeführt. Um die skizzierten Forschungsfragen zu untersuchen, wurden weitere explorative Analysen durchgeführt:

Mögliche Zusammenhänge zwischen den einzelnen Zuschreibungs-Items wurden mittels einer explorativen Faktorenanalyse (Hauptachsenanalyse mit Oblimin-Rotation; [8]) untersucht. Diese Analyse ergab zunächst vier Faktoren, davon zwei mit einem Eigenwert > 1. Auch ein Scree-Plot, eine Parallelanalyse [13] sowie ein Minimal Average Partial Test [27] legten eine Zweifaktorenlösung nahe. Es zeigte sich, dass die sechs inhaltlich positiven Zuschreibungen und die vier inhaltlich negativen Zuschreibungen jeweils substanziell auf einen dieser beiden Faktoren luden (alle λ > 0,45), die gemeinsam 89 % der Varianz erklärten (siehe eTabelle 1 im elektronischen Anhang). Durch Mittelwertbildung wurden zwei Skalen mit akzeptablen internen Konsistenzen gebildet („positive Zuschreibungen“, α = 0,80; „negative Zuschreibungen“, α = 0,68) und für die weiteren Analysen verwendet.

Um mögliche Zusammenhänge zwischen persönlicher Betroffenheit (EKB und deren Offenlegung) einerseits und Haltung zu EKB andererseits genauer zu beleuchten, wurden explorative Gruppenvergleiche durchgeführt. Dafür wurden zunächst die bisherigen Offenlegungserfahrungen dichotomisiert (Offenlegung vs. keine Offenlegung). Anschließend wurden mögliche Unterschiede in den Zuschreibungen gegenüber psychiatrisch Tätigen mit EKB zwischen Teilnehmenden mit und ohne EKB bzw. zwischen den Teilnehmenden mit und ohne Offenlegungserfahrungen mittels einseitigen *t*-Tests untersucht. Auch Unterschiede in der Offenlegungseinstellung zwischen denjenigen Teilnehmenden mit EKB, die ihre EKB (nicht) offengelegt haben, wurden mittels *t*-Tests untersucht.

Alle Auswertungen wurden mit Stata 18 durchgeführt.

## Ergebnisse

### Stichprobe

Insgesamt nahmen 215 Mitarbeiter*innen aus 18 psychiatrischen Abteilungen in Berlin und Brandenburg teil, von denen 182 (84,7 %) angaben, über EKB zu verfügen. Die Berufsgruppen umfassten Ärzt*innen (*n* = 55), Psycholog*innen (*n* = 42), Sozialarbeiter*innen (*n* = 15), Pflegepersonal (*n* = 72) sowie Spezialtherapeut*innen (*n* = 31); der Altersdurchschnitt betrug 41,4 Jahre. Die Datenerhebung erfolgte mittels einer Onlinebefragung, die Fragen zu persönlichen Krisenerfahrungen, Hilfeinanspruchnahme und der Bedeutung dieser Erfahrungen beinhaltete.

Eine Übersicht aller Variablen findet sich in Tab. 1. Eine Übersicht über alle Korrelationen findet sich in eTabelle 2 im elektronischen Anhang.

**Tab. 1:** Deskriptive Kennwerte der Variablen

Items	*M*	*SD*	Med
*Bedeutung: Einschätzung der Effekte von EKB:*			
EKB bei Profis haben einen Einfluss auf die Arbeit mit PatientInnen	3,13	0,76	3,00
Es nützt PatientInnen, wenn Profis über EKB verfügen	2,69	0,91	3,00
*Bedeutung: Zuschreibungen (Items): *Profis mit eigenen Krisen‑/Behandlungserfahrungen …			
haben mehr Empathie	2,25	0,96	2,00
verstehen PatientInnen besser	2,47	0,91	3,00
kennen sich besser mit Gefühlen der Stigmatisierung und Diskriminierung aus	2,60	0,87	3,00
stigmatisieren PatientInnen weniger	2,49	0,98	3,00
vermitteln Hoffnung	2,61	0,81	3,00
fällt es leichter, eine „Beziehung auf Augenhöhe“ mit PatientInnen aufzubauen	2,38	0,99	2,00
sind nicht ausreichend belastbar	1,11	0,94	1,00
können sich schlechter gegenüber PatientInnen abgrenzen	1,61	0,95	2,00
haben mehr „blinde Flecken“ in der Analyse seelischer Krisen	1,35	0,88	1,00
sind stark mit sich selbst beschäftigt	1,27	0,89	2,00
*Bedeutung: Zuschreibungen (aus EFA gebildete Variablen):*			
Positive Zuschreibungen	2,47	0,65	2,50
Negative Zuschreibungen	1,34	0,66	1,25
*Offenlegungseinstellung:*			
Es ist sinnvoll, wenn Profis gegenüber PatientInnen ihre EKB offen thematisieren	1,36	1,06	1,00

*M* Mittelwert, *SD* Standardabweichung*, EFA* explorative Faktorenanalyse *N* = 215

### Wahrgenommene Bedeutung

#### Einschätzung der Auswirkungen von EKB

Die Ergebnisse zeigen eine relativ hohe Zustimmung der Teilnehmenden zu den beiden eingesetzten Items. Der Aussage „Eigene Krisen‑/Behandlungserfahrungen bei Profis haben einen Einfluss auf die Arbeit mit Patient*innen“ stimmten 72,6 % Teilnehmenden eher oder voll und ganz zu, der Mittelwert betrug 3,13, der Median 3,00. Der Aussage, dass es Patient*innen nützte, wenn Profis über EKB verfügen, stimmten 66,1 % eher oder voll und ganz zu, mit einer mittleren Zustimmung von 2,69 und einem Median von 3,00.

#### Zuschreibungen

Die abgefragten positiven Zuschreibungen bezüglich Mitarbeitenden mit EKB fanden durchschnittliche Zustimmungen, die oberhalb des Skalenmittelwerts lagen, sowohl auf Ebene der Einzelitems als auch bezüglich der zusammenfassenden Variable (Tab. 1). Eine Mehrheit der Teilnehmenden schrieb Mitarbeitenden positive Eigenschaften zu (eher oder vollständige Zustimmung), nämlich verständnisvoller gegenüber Patient*innen (56,3 %) und erfahrener mit Gefühlen der Stigmatisierung und Diskriminierung (59,5 %) zu sein, Patient*innen weniger zu stigmatisieren (62,3 %) und ihnen Hoffnung zu vermitteln (60,5 %). Immerhin knapp die Hälfte der Befragten stimmte den Aussagen eher oder vollständig zu, dass Mitarbeitende mit EKB empathischer (45,1 %) sind und es ihnen leichter fällt, Beziehungen „auf Augenhöhe“ mit Patient*innen aufzubauen (49,3 %).

Alle negativen Zuschreibungs-Items wie auch der zusammenfassende Wert zeigten Mittelwerte unterhalb des Skalenmittelpunkts (Tab. 1). Nur ein geringer Teil der Befragten attestierte Mitarbeitenden mit EKB eher oder überwiegend schlechtere Abgrenzung (19,1 %) gegenüber Patient*innen, weniger Belastbarkeit (9,8 %) und mehr „blinde Flecken“ bei der Analyse seelischer Krisen (7,0 %). Am deutlichsten wurde der Aussage zugestimmt, dass Mitarbeitende mit EKB stark mit sich selbst beschäftigt sind (36,8 % eher oder vollständige Zustimmung).

#### EKB und Zuschreibungen

Der Vergleich der Zuschreibungen zwischen Teilnehmenden mit und ohne EKB (*n* = 182 bzw. *n* = 33) deutet darauf hin, dass Befragte mit EKB signifikant mehr positive Eigenschaften bei Mitarbeitenden mit EKB wahrnehmen (*M* = 2,53, *SD* = 0,61 bzw. *M* = 2,12, *SD* = 0,77, *t *[213] = −3,39, *p* < 0,001). Im Gegensatz dazu neigten Befragte ohne EKB dazu, signifikant mehr negative Aussagen zu gewichten (*M* = 1,30, *SD* = 0,66 bzw. *M* = 1,52, *SD* = 0,61, *t *[213] = 1,79, *p* = 0,038).

### Offenlegung

#### Offenlegungseinstellungen

Die Mehrheit der Befragten (57,2 %) lehnte die Aussage eher oder voll und ganz ab, dass es sinnvoll ist, wenn Mitarbeitende ihre EKB gegenüber Patient*innen offenlegen. Nur 14,4 % stimmten eher oder vollständig zu, während 28,4 % eine neutrale Haltung vertraten.

#### Offenlegungspraxis

Gleichzeitig hat eine solche Offenlegung gegenüber Patient*innen in 117 Fällen (64,3 %) noch nie stattgefunden, während sie in 65 Fällen (35,7 %) entweder einmalig oder mehrmals erfolgt ist. Eine Offenlegung hat dabei in 16 Fällen (8,8 %) einmalig stattgefunden, in 43 Fällen (23,6 %) mehrmals, in 3 Fällen (1,7 %) regelmäßig und in weiteren 3 Fällen (1,7 %) häufig.

#### Offenlegungspraxis und Zuschreibungen

Der Vergleich von Befragten mit EKB mit und ohne Offenlegungserfahrungen (*n* = 65 bzw. *n* = 117) ergab, dass die Gruppe mit Offenlegung signifikant mehr positive Eigenschaften bei Mitarbeitenden mit EKB wahrnahm (*M* = 2,70, *SD* = 0,58 bzw. *M* = 2,44, *SD* = 0,61, *t *[180] = −2,82, *p* = 0,003). Hingegen gab es keinen signifikanten Unterschied in der Wahrnehmung negativer Eigenschaften zwischen den beiden Gruppen (*M* = 1,29, *SD* = 0,67 bzw. *M* = 1,31, *SD* = 0,66, *t *[180] = 0,15, *p* = 0,560).

#### Offenlegungspraxis und Offenlegungseinstellungen

Teilnehmende mit EKB, die diese gegenüber Patient*innen offengelegt haben, betrachteten Offenlegung als signifikant sinnvoller als diejenigen Teilnehmenden ohne bisherige Offenlegung (*M* = 1,88, *SD* = 0,99 bzw. *M* = 1,03, *SD* = 0,97, *t *[180] = −5,56, *p* < 0,001).

## Diskussion

Zusammenfassend erkennen die befragten Mitarbeitenden die hohe Bedeutung von EKB in ihrer patient*innenbezogenen Arbeit an. Eine Mehrheit ist der Meinung, dass diese Erfahrungen einen Einfluss auf ihre Tätigkeit haben und auch, dass es Patient*innen nutzt, wenn Mitarbeitende selbst über EKB verfügen. Auch die insgesamt hohe Zustimmung zu den positiven Zuschreibungen und geringe Zustimmung zu den negativen Zuschreibungen zeigen an, dass EKB im Patient*innenkontakt als bedeutsam eingeschätzt wird, eine Einschätzung, die bei Befragten, die sich selbst als betroffen einschätzen, sogar noch weiter ansteigt. Trotz dieser insgesamt hohen Bedeutung, die EKB beigemessen wird, legen die befragten Mitarbeitenden diese Erfahrungen nur selten offen bzw. halten von einer solchen Offenlegung nur wenig: Die meisten halten es nicht für sinnvoll, EKB gegenüber Patient*innen zu offenbaren und praktizieren eine solche Offenlegung auch nicht. Gleichzeitig sind diejenigen, die EKB offengelegt haben, einer Offenlegung positiver gegenüber eingestellt und schreiben EKB auch einen höheren Nutzen zu.

### Hohe Bedeutung von EKB …

Auch in der Literatur wird EKB ein hoher Nutzen zugeschrieben. Hier sind vor allem Arbeiten zur Peer- und Genesungsbegleitung zu nennen, die inzwischen einen hohen Grad an Evidenz für die Wirksamkeit von Erfahrungswissen im patient*innenbezogenen Kontakt aufbietet. Sowohl in kontrollierten Studien als auch in systematischen Reviews sowie in Arbeiten, die qualitativ-narrative Evidenz aufbereiten, zeigt sich, dass der reflektierte Einsatz von Erfahrungswissen von Peer- und Genesungsbegleiter*innen dazu führt, dass Patient*innen mehr Selbstwirksamkeit entwickeln und sich selbst weniger stigmatisieren [17, 31]. Darüber hinaus stärkt es die Selbstexploration der Nutzer*innen und reduziert ihre Symptombelastung [32]. Im Kontext der Peer- und Genesungsbegleitung wird es dabei als wichtig erachtet, dass gemachte Krisen- und Behandlungserfahrungen der Peer- und Genesungsbegleitenden kollektiv – bspw. im Rahmen von „ExIn“-Kursen oder Genesungsgruppen – reflektiert werden und damit anschlussfähig auch für andere Krisen- und Genesungsgeschichten werden können [6, 10].

Demgegenüber fehlen systematischere Untersuchungen zum Nutzen und zur Wirksamkeit von EKB im Fall von Mitarbeitenden, die in psychiatrischen oder psychosozialen Einrichtungen nicht als Peer- und Genesungsbegleiter*innen arbeiten, noch weitgehend. Die wenigen Arbeiten, die sich diesem Thema zuwenden, machen deutlich, dass Mitarbeiter*innen mit EKB durch ihr tiefes Verständnis und ihre Empathie eine verbesserte Patientenbetreuung gewährleisten. Sie fördern Vertrauen und reduzieren Stigmatisierung. Ihre Erfahrungen bereichern das Team, stärken ihre berufliche Identität und tragen zur persönlichen Resilienz bei [3, 20].

Dieser Nutzen wird in vielen Arbeiten vor allem von den Mitarbeitenden mit EKB selbst gesehen, bspw. in Arbeiten, die ein autoethnographisches Design nutzen [9, 15]. Ähnlich verhält es sich mit den vorliegenden Ergebnissen: Befragte, die selbst über EKB verfügten, äußerten sich insgesamt positiver in Bezug auf die Bedeutung dieser Erfahrungen. Sie schrieben Mitarbeitenden mit EKB mehr positive und weniger negative Eigenschaften zu und zeigen damit an, dass sie diese Mitarbeitenden-Gruppe im patient*innenbezogenen Kontakt für kompetent(er) halten. Zusammenfassend heißt das, dass Mitarbeitenden mit EKB durch die Befragten und in der Literatur spezifische Kompetenzen zugeschrieben werden, über die Mitarbeitende ohne EKB möglicherweise nicht verfügen. Dies sehen in der vorliegenden Studie zwar nicht alle Befragten so – ein kleiner Anteil schätzt diese Mitarbeitenden-Gruppe als weniger kompetent ein – aber doch der überwiegende Teil.

### … und trotzdem geringe Offenlegung

Angesichts dessen überrascht, dass die Befragten ihre EKB trotz grundsätzlicher Zustimmung ihrer Bedeutung und ihres Nutzens nur selten offenlegen bzw. von einer solchen Offenlegung gegenüber Patient*innen nur wenig halten. Dieser Befund zeigt sich auch in anderen Studien [4] und wird auch in der bereits in der Einleitung erwähnten zweiten Arbeit aus der EKBM-Studie zur Frage der Offenlegung, in diesem Fall gegenüber Kolleg*innen, diskutiert [28]. Sie zeigt, dass Mitarbeitende ihre EKB bereits im Kreis ihrer Kolleg*innen nur eingeschränkt und selektiv offengelegen: Zwar hatten fast zwei Drittel ihre EKB gegenüber Kolleg*innen offengelegt, dies aber vor allem gegenüber nahestehenden Kolleg*innen oder gegenüber Vorgesetzten, was darauf hinweist, dass für eine Offenlegung entweder Vertrauen notwendig ist oder diese nur oder vor allem dann erfolgt, wenn sie unvermeidbar ist, bspw. im Zusammenhang mit Krankenschreibungen oder zur Anpassung von Arbeitsverhältnissen.

Gegenüber Patient*innen sind die befragten Mitarbeitenden noch vorsichtiger: Während ca. ein Drittel der Mitarbeitenden ihre EKB gegenüber Kolleg*innen noch nie offengelegt hat, haben fast zwei Drittel der Befragten diese Erfahrungen noch nie gegenüber Patient*innen offengelegt, also etwa doppelt so häufig. Um diese große Zurückhaltung zu verstehen, finden sich in der Literatur einige Anhaltspunkte: Mitarbeitende mit EKB werden vor allem unter Kolleg*innen (vs. durch Patient*innen) erheblich stigmatisiert [24], was Grund genug für eine Zurückhaltung in diesem Kreis sein kann. Konzepte wie „therapeutische Abstinenz“ [2] oder eine der psychiatrieimmanenten Aufteilung in „gesunde Mitarbeitende“ einerseits und „kranke Patient*innen“ andererseits [7] können eine solche Vorsicht gegenüber Patient*innen begründen. Eine Konstruktion im „dazwischen“ dieser beiden Gruppen erfordert harte reflexive und emotionale Arbeit [22], die nicht jede*r Mitarbeitende*r bereit ist, zu leisten. Einmal „geoutet“, ist diese Arbeit oft zur alltäglichen Aufgabe geworden, sodass eine Offenlegung beiden Gruppen gegenüber als „logisch“ erlebt wird, insbesondere, weil eine Geheimhaltung von EKB von vor allem bereits geouteten Mitarbeitenden als sehr anstrengend erlebt wird [9, 14].

Ein weiteres Paradox der EKB-Studie könnte außerdem als Erklärung herangezogen werden: Die befragten Mitarbeitende haben sich selbst wenig bis gar nicht stigmatisiert [26]. Was auf den ersten Blick positiv erscheint, wirft auf den zweiten Blick Fragen auf: Wie schaffen die Befragten es, trotz teils einschneidender Erfahrungen erheblicher Krisen und mit diversen Behandlungsformen, das damit üblicherweise verbundene Stigma abzuwehren? Im Zusammenhang mit dem Befund der Nichtoffenlegung – vor allem gegenüber Patient*innen, aber auch gegenüber Kolleg*innen – drängt sich der Verdacht auf, dass diese dazu dient, mit Stigma zurechtzukommen bzw. Stigma im Sinne einer Resilienz [16] abzuwehren. Werden EKB also weniger aufgrund einer Abstinenz oder anderer therapeutischer Logiken nicht offengelegt, sondern eher im Sinne einer, sicher oft unbewussten, Strategie, das mit EKB üblicherweise verbundene Stigma von sich abzuwenden?

### Kann EKB ohne Offenlegung wirken?

Wie oben genannt, wird die Wirksamkeit der Peer- und Genesungsbegleitung oft in den Zusammenhang mit kollektiv reflektierten Krisen- und Behandlungserfahrungen gebracht. Wie stark diese Wirksamkeit auch von der Offenlegung seitens der Peer- und Genesungsbegleitenden abhängt, ist weniger systematisch im Fokus. In Arbeiten zu autonomen, selbst organisierten Formen des Peer-Supports ist eine solche Offenlegung unhinterfragt [5, 6]. In mehreren Übersichtsarbeiten [31, 32] wird eine solche Offenlegung als zentraler Bestandteil der Peer- und Genesungsbegleitung aufgegriffen. Und auch in einer Programmtheorie [10] wird ein „reflektierter Umgang“ mit EKB gegenüber Patient*innen als zentraler Bestandteil der Peer- und Genesungsbegleitung beschrieben. Wie explizit sich Peer- und Genesungsbegleiter*innen in ihrer alltäglichen Arbeit (immer) auf ihre EKB beziehen, kann nur schwer beantwortet werden. In jedem Fall erscheint es als Widerspruch, auf der Grundlage von Erfahrungswissen zu arbeiten, wenn dieses nicht offengelegt oder geteilt wird.

So haben Peer- und Genesungsbegleitende oft keine Wahl, ihre Erfahrungen offenzulegen bzw. versteckt zu halten. Sie werden auf der Grundlage dieser Erfahrungen eingestellt und werden schon aufgrund ihrer Tätigkeitsbezeichnung regelmäßig mit Offenlegung konfrontiert. Das heißt auch, dass sie mit dem Stigma, das mit einer Offenlegung verbunden ist, immer wieder umgehen müssen, nicht nur in der Gesellschaft, sondern auch gegenüber Mitarbeitenden und Patient*innen in der Behandlungssituation. Dadurch können sie hilfreiche Strategien zum Umgang mit Stigma an Patient*innen weitergeben, was durch verschiedene Studien auch belegt ist [32]. Ob solche Strategien auch von Mitarbeitenden vermittelt werden können, die ihre EKB nicht offenlegen, bleibt fraglich. Die Einschätzung der Befragten unserer Studie, dass Mitarbeitende mit EKB erfahrener im Umgang mit Stigmatisierung und weniger stigmatisierend sind, muss angesichts des genannten Befunds der geringen Selbststigmatisierung kritisch hinterfragt werden: Trifft diese Einschätzung auf alle Mitarbeitende mit EKB zu oder nur auf diejenigen, die diese Erfahrungen selbst auch offenlegen bzw. auch selbst mit Stigma ringen?

Übergeordneter führt diese Diskussion zu der Frage, ob nichtoffengelegte EKB in derselben Weise wirken können wie offengelegte Erfahrungen: Ist eine Offenlegung nicht ein notwendiger Bestandteil dafür, dass EKB auch für Patient*innen nutzbringend eingebracht werden (können)? Kommt der von den Befragten zugeschriebene hohe Nutzen von EKB in der therapeutischen Situation tatsächlich in die Anwendung, wenn diese Erfahrungen nicht offengelegt werden? Zu diesen Fragen sind Anschlussstudien erforderlich. An dieser Stelle möchten wir zumindest die Hypothese wagen, dass nichtoffengelegte EKB von Mitarbeitenden psychiatrischer Einrichtungen nicht in derselben Weise wirksam werden können wie offengelegte Erfahrungen – eine Hypothese, die auch durch ein weiteres Ergebnis unserer Studie unterstützt wird: Diejenigen Mitarbeitenden, die ihre EKB offengelegt haben, schreiben Mitarbeitenden mit EKB positivere Eigenschaften zu und haben (oder entwickelten?) in Bezug auf Offenlegung auch eine positivere Einstellung. Die Offenlegung von EKB scheint in der patient*innenorientierten Arbeit also als nutzbringend erlebt zu werden.

## Limitationen

Der Selektionsbias der Teilnahme an der Studie ist bereits in der Hauptpublikation ausführlich diskutiert worden [29]: Vermutlich haben vor allem Mitarbeitende mit EKB an der Studie teilgenommen und darunter vor allem auch diejenigen, die ihre EKB offenlegen, sodass das Vorkommen dieser Erfahrungen, ihre Offenlegung, und ihre positive Bewertung überschätzt werden. Durch die explorative Natur der Studie wurden für die Erhebung keine standardisierten Instrumente genutzt. Alle Maße sind Selbstberichte, die vielleicht durch Erinnerungseffekte beeinflusst sind, wie Verklärung oder Dramatisierung im Nachhinein. Auch sollten aufgrund der explorativen Anlage alle inferenzstatistischen Ergebnisse vorsichtig interpretiert werden. Und schließlich nutzten wir ein Querschnittdesign, sodass keine Kausalattribution möglich sind, was v. a. in Bezug auf die Offenlegungsgründe ins Gewicht fällt.

## Schlussfolgerungen

Aus all dem, was wir von der Peer- und Genesungsbegleitung wissen, ist anzunehmen, dass EKB gegenüber Patient*innen offen kommuniziert werden müssen, damit sie wirksam werden können. Vermutlich reicht es nicht, dass Fachpersonal psychiatrischer oder psychosozialer Einrichtungen die Bedeutung von EKB für die patient*innenbezogene Arbeit hoch schätzen, sondern sie müssen diese Erfahrungen auch offenlegen. Das heißt nicht, dass sie Patient*innen mit diesen Erfahrungen belasten sollen. Stattdessen sollten EKB dosiert und abgewogen, im Sinne der „reflektierten Offenlegung“ der Peer- und Genesungsbegleitung, in die patient*innenbezogene Arbeit eingebracht werden. In Vorbereitung darauf benötigt es Austauschräume und Ressourcen, wie das beispielsweise die Gruppe von selbst betroffenen Profis der Deutschen Gesellschaft für Bipolare Störungen [1] schon seit Jahren tut. Ähnliche Initiativen sind auf bundesweiter und lokaler Ebene notwendig, um den Nutzen von EKB bei Mitarbeitenden psychiatrischer und psychosozialer Einrichtungen wirklich ausschöpfen zu können.

## Supplementary Information

eTabelle 1 Explorative Faktorenanalyse der Zuschreibungs-Items mit Oblimin-Rotation

eTabelle 2 Übersicht über alle gebildeten Korrelationen

## Data Availability

Alle Daten, die die Ergebnisse dieser Studie untermauern, sind in der Veröffentlichung und in den ergänzenden Informationen verfügbar. Aus Gründen der Sensibilität sind nicht alle Daten, die die Ergebnisse dieser Studie stützen, öffentlich zugänglich, können aber auf begründete Anfrage beim entsprechenden Autor angefordert werden.
