# Supplementary material for: Psychiatric professionals’ own crisis and treatment experiences—impact and coping strategies
Source: Nervenarzt. 2025 Mar 14;97(1):67–73. [Article in German] doi: 10.1007/s00115-025-01815-9 (PMC12808170; doi:10.1007/s00115-025-01815-9)
Supplement: Supplementary file 1 — eTabelle 1 Explorative Faktorenanalyse der Zuschreibungs-Items mit Oblimin-Rotation [file 115_2025_1815_MOESM1_ESM.docx]

**eTabelle 1:** Explorative Faktorenanalyse der Zuschreibungs-Items mit Oblimin-Rotation

|  |  | Rotierte Faktorladungen | | | |
| --- | --- | --- | --- | --- | --- |
|  | Items (Profis mit EKB …) | Faktor 1 | Faktor 2 | Faktor 3 | Faktor 4 |
|  | … haben mehr Empathie | .79 |  |  |  |
|  | … verstehen PatienInnen besser | .76 |  |  |  |
|  | … kennen sich besser mit Gefühlen der Stigmatisierung und Diskriminierung aus | .49 |  | -.24 |  |
|  | … stigmatisieren PatientInnen weniger | .57 |  |  | .25 |
|  | … vermitteln Hoffnung | .51 |  |  | .24 |
|  | … fällt es leichter, eine „Beziehung auf Augenhöhe“ mit PatientInnen aufzubauen | .71 |  |  |  |
|  | … sind nicht ausreichend belastbar |  | .64 |  |  |
|  | … können sich schlechter gegenüber PatientInnen abgrenzen |  | .56 |  |  |
|  | … haben mehr „blinde Flecken“ in der Analyse von seelischen Krisen |  | .46 | .29 |  |
|  | … sind stark mit sich selbst beschäftigt |  | .64 |  |  |

*Anmerkung: N* = 215. Faktorladungen < .20 werden nicht dargestellt.
