# Supplementary material for: Psychiatric professionals’ own crisis and treatment experiences—impact and coping strategies
Source: Nervenarzt. 2025 Mar 14;97(1):67–73. [Article in German] doi: 10.1007/s00115-025-01815-9 (PMC12808170; doi:10.1007/s00115-025-01815-9)
Supplement: Supplementary file 2 — eTabelle 2 Übersicht über alle gebildeten Korrelationen [file 115_2025_1815_MOESM2_ESM.docx]

**eTabelle 2:** Übersicht über alle gebildeten Korrelationen

|  | Variable | 1 | 2 | 3 | 4 | 5 | 6 | 7 | 8 | 9 | 10 | 11 | 12 | 13 | 14 | 15 | 16 |
| --- | --- | --- | --- | --- | --- | --- | --- | --- | --- | --- | --- | --- | --- | --- | --- | --- | --- |
| 1 | EKB bei Profis haben einen Einfluss auf die Arbeit mit PatientInnen | 1 | .37*** | .23*** | .27*** | .22** | .29*** | .25*** | .29*** | -.18* | -.09 | -.21** | -.09 | .16 | .19** | .04 | -.06 |
| 2 | Es nützt PatientInnen, wenn Profis über EKB verfügen |  | 1 | .39*** | .42*** | .19** | .26*** | .30*** | .32*** | -.22** | -.10 | -.11 | -.19* | .30*** | .27*** | .09 | .02 |
| 3 | Profis mit EKB haben mehr Empathie |  |  | 1 | .60*** | .38*** | .39*** | .34*** | .48*** | .03 | .00 | .14 | -.00 | .21** | .12 | .05 | -.06 |
| 4 | Profis mit EKB verstehen PatienInnen besser |  |  |  | 1 | .29*** | .42*** | .33*** | .60*** | -.10 | -.04 | .04 | -.06 | .21** | .24*** | .09 | -.07 |
| 5 | Profis mit EKB kennen sich besser mit Gefühlen der Stigmatisierung und Diskriminierung aus |  |  |  |  | 1 | .33*** | .31*** | .39*** | .06 | .02 | -.03 | .05 | .25*** | .10 | -.06 | -.03 |
| 6 | Profis mit EKB stigmatisieren PatientInnen weniger |  |  |  |  |  | 1 | .39*** | .38*** | .00 | .08 | .20** | .04 | .18* | .17 | .07 | -.03 |
| 7 | Profis mit EKB vermitteln Hoffnung |  |  |  |  |  |  | 1 | .39*** | -.12 | -.09 | -.07 | -.15 | .29*** | .29*** | .17 | -.08 |
| 8 | Profis mit EKB fällt es leichter, eine „Beziehung auf Augenhöhe“ mit PatientInnen aufzubauen |  |  |  |  |  |  |  | 1 | .00 | .01 | .04 | -.01 | .36*** | .29*** | .02 | .04 |
| 9 | Profis mit EKB sind nicht ausreichend belastbar |  |  |  |  |  |  |  |  | 1 | .34*** | .33*** | .48*** | -.06 | -.03 | -.00 | .06 |
| 10 | Profis mit EKB können sich schlechter gegenüber PatientInnen abgrenzen |  |  |  |  |  |  |  |  |  | 1 | .31*** | .42*** | -.03 | -.14 | -.11 | .06 |
| 11 | Profis mit EKB haben mehr „blinde Flecken“ in der Analyse von seelischen Krisen |  |  |  |  |  |  |  |  |  |  | 1 | .23*** | .04 | -.03 | .07 | .00 |
| 12 | Profis mit EKB sind stark mit sich selbst beschäftigt |  |  |  |  |  |  |  |  |  |  |  | 1 | -.08 | -.08 | -.14 | .16 |
| 13 | Es ist sinnvoll, wenn Profis gegenüber PatientInnen ihre EKB offen thematisieren |  |  |  |  |  |  |  |  |  |  |  |  | 1 | .46*** | .01 | .11 |
| 14 | Berichtete Offenlegung gegenüber PatientInnen |  |  |  |  |  |  |  |  |  |  |  |  |  | 1 | .21** | .01 |
| 15 | Alter^a^ |  |  |  |  |  |  |  |  |  |  |  |  |  |  | 1 | -.01 |
| 16 | Geschlecht |  |  |  |  |  |  |  |  |  |  |  |  |  |  |  | 1 |

*Anmerkung: N* = 215.

^a^*n* = 211. ^b^*n* = 214; 0 = weiblich, 1 = männlich, 2 = divers.

**p* < .010, ***p* < .005, ****p* < .001.
